# Bridging Cardiac and Mental Health: Screening for Depression in Cardiology Settings

**DOI:** 10.7759/cureus.102107

**Published:** 2026-01-22

**Authors:** Sumit Kumar, Arijita Banerjee

**Affiliations:** 1 Psychiatry and Behavioral Sciences, Indian Center for Advancement of Research and Education (ICARE) Institute of Medical Sciences and Research, Haldia, IND; 2 Physiology, Indian Institute of Technology Kharagpur, Kharagpur, IND

**Keywords:** cardiovascular disease, collaborative care model, depression screening, patient health questionnaire-2, patient health questionnaire-9, patient health questionnaire (phq-9)

## Abstract

Depression is a highly prevalent comorbidity in patients with cardiovascular disease (CVD), affecting approximately one in five cardiac patients and contributing to adverse cardiovascular outcomes, increased mortality, and reduced quality of life. Despite clinical guidelines from the American Heart Association recommending routine depression screening, implementation remains inconsistent across cardiology settings. This narrative review synthesizes current evidence on depression screening practices in cardiology clinics, examining prevalence, screening tools, and implementation barriers. The prevalence of depression in cardiovascular patients ranges from 15% to 30%, with higher rates observed in post-myocardial infarction and heart failure populations globally. The two-step screening approach using Patient Health Questionnaire-2 (PHQ-2) followed by Patient Health Questionnaire-9 (PHQ-9) demonstrates optimal sensitivity (83-87%) and specificity (78-92%) while minimizing patient burden. Major implementation barriers include time constraints, inadequate workflow integration, insufficient mental health resources, provider knowledge gaps, and patient-level factors, including stigma and poor treatment acceptance. Successful implementation of depression screening in cardiology clinics requires multilevel strategies addressing system, provider, and patient factors through electronic health record integration, standardized protocols, collaborative care models, and stakeholder engagement.

## Introduction and background

Cardiovascular disease (CVD) remains the leading cause of morbidity and mortality globally, with ischemic heart disease and stroke affecting millions of individuals annually. Among patients with CVD, depression emerges as a critical but often under-recognized comorbidity that significantly impacts both cardiac prognosis and quality of life. The bidirectional relationship between depression and cardiovascular disease has been well-established through decades of research, with depression serving both as a risk factor for developing CVD and as a consequence of cardiac events [1,2].

The estimated global prevalence of depression among cardiovascular patients is approximately 20.8%, with specific rates of 19.8% in coronary artery disease and 24.7% in heart failure populations. The diagnostic criteria for major depressive disorder require the presence of five or more symptoms (including depressed mood or loss of interest/pleasure, plus additional symptoms like sleep disturbances, appetite changes, fatigue, feelings of worthlessness, concentration difficulties, or suicidal thoughts) occurring nearly every day for at least two weeks and causing significant functional impairment, as outlined in the Diagnostic and Statistical Manual of Mental Disorders, Fifth Edition (DSM-5). This prevalence is substantially higher than the general population rate of approximately 7%, highlighting the vulnerability of cardiac patients to depressive disorders. Depression occurs approximately three times more frequently in patients following acute myocardial infarction compared to the general community, with 15-20% of post-myocardial infarction patients meeting diagnostic criteria for major depression [3,4].

The clinical significance of depression in cardiac patients extends far beyond mental health concerns. A graded relationship exists between depression severity and subsequent cardiovascular mortality risk, with more severe depression associated with higher rates of adverse events. Depression in CVD patients contributes to non-adherence to cardiac medications, reduced participation in cardiac rehabilitation, unhealthy lifestyle behaviors including poor diet and physical inactivity, and increased healthcare utilization and costs [5,6].

Pathophysiological mechanisms

The relationship between depression and cardiovascular disease involves multiple interconnected pathophysiological pathways. Autonomic nervous system dysfunction, characterized by reduced heart rate variability and increased sympathetic activity, represents a primary mechanism linking depression to adverse cardiac outcomes. Autonomic dysregulation is central to both conditions. Depression and anxiety increase sympathetic nervous system activity while reducing parasympathetic tone, leading to elevated heart rate, reduced heart rate variability, and increased risk of arrhythmias. This autonomic imbalance contributes to worse cardiovascular outcomes [7].

Hypothalamic pituitary axis (HPA) dysfunction occurs in both depression and cardiovascular disease. Chronic stress and depression cause sustained cortisol elevation, which promotes atherosclerosis, endothelial dysfunction, hypertension, and metabolic syndrome. The hyperactive stress response also increases cardiovascular reactivity to acute stressors. Inflammatory mechanisms link these conditions through elevated pro-inflammatory cytokines (IL-6, tumor necrosis factor-alpha {TNF-α}, CRP). Depression is associated with chronic low-grade inflammation that accelerates atherosclerosis and plaque instability. The inflammation also affects neurotransmitter metabolism, creating a vicious cycle [8].

Platelet activation and coagulation abnormalities are more pronounced in depressed cardiac patients, with increased platelet aggregation, higher levels of clotting factors, and reduced fibrinolysis. This creates a prothrombotic state, increasing myocardial infarction (MI) and stroke risk. Endothelial dysfunction occurs through multiple mechanisms, including reduced nitric oxide availability, increased oxidative stress, and direct vascular effects of elevated catecholamines and cortisol [9].

Behavioral and psychosocial factors also contribute significantly to the depression-CVD relationship. Depressed cardiac patients demonstrate lower rates of medication adherence, reduced engagement in cardiac rehabilitation programs, continued tobacco use, poor dietary habits, and social isolation - all of which independently predict worse cardiovascular outcomes [10,11].

Clinical practice guidelines

Growing awareness of the bidirectional link between depression and cardiovascular disease has led major cardiac societies to introduce formal screening guidelines. The U.S. Preventive Services Task Force (USPSTF) identifies adults with CVD as a high-risk group and advises routine screening with established, validated tools. Similarly, the American Heart Association recommends administering the Patient Health Questionnaire-2 (PHQ-2) to all individuals with coronary artery disease, followed by the Patient Health Questionnaire-9 (PHQ-9) when initial results are positive [12]. Screening is advised not only during hospitalization but also at consistent intervals after a myocardial infarction. The European Society of Cardiology echoes these recommendations, particularly emphasizing routine depression assessment in patients living with heart failure. However, despite these guidelines, a recent systematic analysis of 65 cardiovascular clinical practice guidelines found that only 37% provided depression screening recommendations, 34% included treatment recommendations, and merely 23% addressed both screening and treatment. This highlights a significant gap between guideline development and comprehensive implementation guidance [13,14]. The present narrative review synthesizes current evidence on depression screening practices in cardiology clinics, examining prevalence, screening tools, and implementation barriers.

## Review

Methods

Search Strategy and Selection Criteria

**Figure 1:**
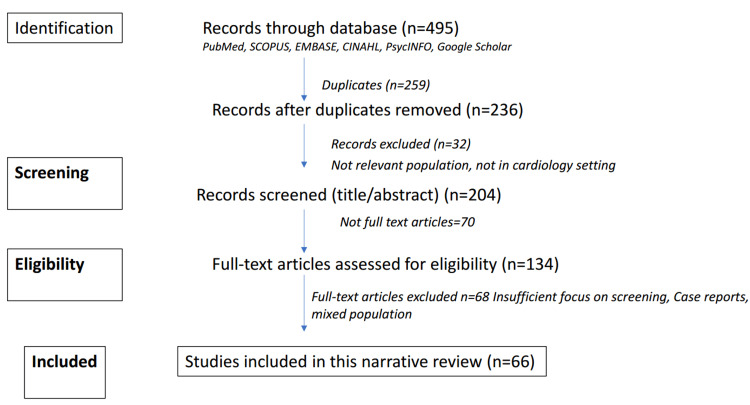
PRISMA flow diagram: depression in cardiac populations. PRISMA: Preferred Reporting Items for Systematic Reviews and Meta-Analyses

This narrative review was conducted in accordance with the Preferred Reporting Items for Systematic Reviews and Meta-Analyses (PRISMA) guidelines for transparency and comprehensiveness (Figure 1). We searched multiple electronic databases, including PubMed, Scopus, Embase, CINAHL, PsycINFO, and Google Scholar, for articles published between January 2015 and September 2025. The inclusion of studies from 2015 to 2025 ensures the incorporation of contemporary research advances in understanding the bidirectional relationship between depression and cardiovascular disease, including refined diagnostic criteria with the implementation of the Diagnostic and Statistical Manual of Mental Disorders, Fifth Edition (DSM-5), which have been elucidated with greater clarity in recent years. The search strategy employed combinations of keywords including “depression screening,” “cardiovascular disease,” “cardiology clinic,” “Patient Health Questionnaire,” “PHQ-2,” “PHQ-9,” “Depression scales,” “coronary heart disease,” “myocardial infarction,” “heart failure,” “implementation,” and “barriers.”

Inclusion and Exclusion Criteria

Studies were included if they (1) focused on depression screening in adult cardiac populations, (2) were conducted in cardiology clinic settings or involved cardiovascular patients, (3) examined screening tools, implementation strategies, or clinical outcomes, (4) were published in English, and (5) represented original research, systematic reviews, meta-analyses, or clinical guidelines. Exclusion criteria included the following: studies focusing solely on pediatric populations, case reports, editorials without original data, and studies not primarily addressing depression screening in cardiac populations. The exclusion of pediatric populations from this review is based on fundamental developmental, epidemiological, and clinical distinctions that warrant separate examination.

Data Extraction and Synthesis

Data were extracted regarding study design, population characteristics, screening instruments utilized, prevalence of positive screens, implementation strategies, identified barriers and facilitators, clinical outcomes, and recommendations. Because the included studies varied widely in their methods and reported outcomes, a narrative synthesis was used to collate and interpret the evidence across them.

Results

This PRISMA flow diagram illustrates the systematic literature search and selection process for this narrative review on depression screening in cardiology settings. The initial database search across PubMed, Scopus, Embase, CINAHL, PsycINFO, and Google Scholar yielded 495 records, which were reduced to 236 after removing 259 duplicates. Following title and abstract screening that excluded 32 records for not meeting population or setting criteria and 70 records without full-text availability, 134 full-text articles were assessed for eligibility. After excluding 68 articles due to insufficient focus on screening tools, case report format, or mixed population studies, 66 studies were ultimately included in this narrative review. While all 66 studies informed the synthesis and conclusions of this review, the reference list includes only the most relevant and representative studies that directly support the key findings and recommendations presented, as including all studies would be impractical for a narrative review format and potentially reduce readability without substantially enhancing the evidence base for the primary conclusions.

Depression Prevalence and Clinical Outcomes in Cardiac Populations

**Figure 2:**
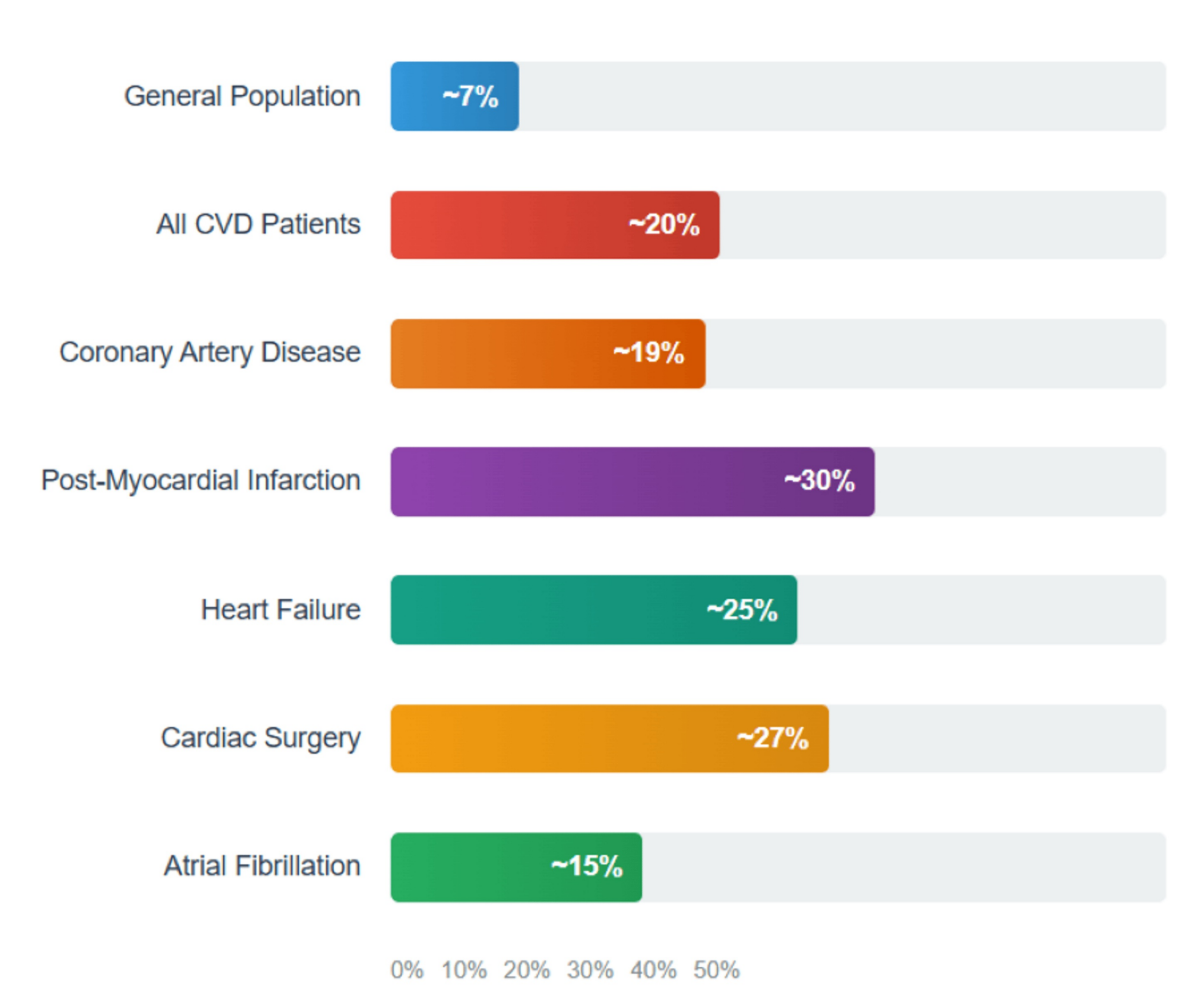
Depression prevalence in cardiac populations. CVD: cardiovascular disease

The global burden of depression varies across different cardiac conditions and clinical contexts (Figure 2) [14]. Meta-analytic evidence indicates that approximately 20% of patients with cardiovascular diseases experience depression. However, prevalence rates demonstrate considerable variation depending on the specific cardiac condition, timing of assessment, and screening methodology employed. Post-myocardial infarction patients represent a particularly high-risk group. Approximately one in five individuals who experience a heart attack is found to have depression soon after the event, with at least equivalent prevalence in heart failure patients. The timing of assessment significantly influences detection rates, with some studies documenting prevalence rates as high as 30-40% when comprehensive screening protocols are implemented [14-16].

The presence of depression in cardiac patients substantially worsens clinical outcomes across multiple domains. Patients diagnosed with cardiovascular disease and comorbid depression face heightened mortality risk within a five-year timeframe, with prognosis remaining unsatisfactory even after treatment targeting a single disorder. Depression contributes to a 26% increase in adverse cardiovascular events, including recurrent myocardial infarction, heart failure hospitalization, and cardiovascular mortality. Quality of life represents another critical outcome domain affected by depression. Depressed cardiac patients consistently report lower physical and mental health-related quality of life scores compared to non-depressed counterparts. The impact extends to functional capacity, with depression associated with reduced exercise tolerance, decreased participation in daily activities, and impaired social functioning [17,18].

Screening Instruments and Psychometric Properties

The Patient Health Questionnaire series represents the most widely recommended and validated screening instruments for depression in cardiac populations (Table 1) [19-24]. The two-step screening process recommended by the American Heart Association (AHA) begins with the PHQ-2, followed by a focused assessment with the PHQ-9 for those who screen positive, minimizing burden while systematically assessing depressive symptoms, including suicidality. The PHQ-2 demonstrates sensitivity of 83% and specificity of 92% at the recommended cutoff score of 3 or greater. The PHQ-9 shows good diagnostic performance with sensitivity ranging from 0.83 to 0.87 and specificity from 0.78 to 0.92 in most primary care and medical populations, though performance may vary in specific cardiac populations [19,20].

**Table 1:** Characteristics of common depression screening instruments in cardiology settings. PHQ-2: Patient Health Questionnaire-2; PHQ-9: Patient Health Questionnaire-9; HADS: Hospital Anxiety and Depression Scale; BDI: Beck Depression Inventory; CES-D: Center for Epidemiologic Studies Depression Scale; AHA: American Heart Association; DSM-5: Diagnostic and Statistical Manual of Mental Disorders, Fifth Edition; CVD: cardiovascular disease

Instrument	Items	Time to complete	Cutoff score	Sensitivity	Specificity	Advantages	Limitations
PHQ-2	2	<1 minute	≥3	83%	92%	Ultra-brief, excellent first-line screen, easy to administer, and AHA-recommended	Requires PHQ-9 follow-up for positive screens
PHQ-9	9	2-3 minutes	≥10	83-87%	78-92%	AHA recommended, includes suicide item, DSM-5 aligned, free, and public domain	Optimal cutoff varies by population and may need adjustment in cardiac patients
HADS	14	5 minutes	≥8	80%	87%	Excludes somatic symptoms, separate anxiety/depression subscales, and validated in cardiac populations	Longer administration time, interpretation more complex, and not DSM-aligned
BDI-II	21	5-10 minutes	≥14	81%	92%	Extensive validation, comprehensive symptom coverage, and well-established	Length limits feasibility, cognitive demands, and requires licensing fee
CES-D	20	5-10 minutes	≥16	84%	79%	Good validation in CVD populations, public domain, and widely used in research	Length includes some somatic items and lower specificity

In coronary artery disease patients specifically, optimal sensitivity (76%) and specificity (76%) have been demonstrated at PHQ-9 cutoff scores of 6 or greater, with an area under the receiver operating characteristic (ROC) curve of 0.86. The variability in optimal cutoff points across populations underscores the importance of validation studies in specific cultural and clinical contexts. While the PHQ series represents the gold standard recommended by major cardiovascular organizations, alternative instruments have been validated in cardiac populations. The Hospital Anxiety and Depression Scale (HADS), Beck Depression Inventory (BDI), and Center for Epidemiologic Studies Depression (CES-D) Scale have all demonstrated acceptable psychometric properties. However, the PHQ instruments offer advantages, including brevity, ease of administration, free availability, public-domain status, and specific recommendations from cardiovascular guidelines [21-23].

The recommended two-step paradigm involves presenting all patients with the PHQ-2, with the questionnaire immediately expanding to include the additional seven items of the PHQ-9 for those screening positive. This approach optimizes efficiency while maintaining diagnostic accuracy. The PHQ-9 item addressing suicidal ideation provides critical safety information requiring immediate clinical response when endorsed [24].

Electronic Health Record Integration

Implementation fidelity studies reveal significant gaps between intended and actual screening practices. In one study of 1,170 coronary heart disease patients, 74.1% were screened at least once over multiple visits, but only 2.5% had positive PHQ-2 screens documented, with 76.2% of those receiving follow-up PHQ-9 assessment. Notably, high-fidelity research-administered screening identified substantially higher rates of positive screens (15-40%), suggesting significant under-detection in routine clinical practice [25]. Routine screening requires minimal time and resources in cardiovascular settings but necessitates increased downstream support from mental health clinicians as depression is more frequently identified. Collaborative care models that integrate mental health professionals into cardiology practices represent evidence-based approaches to addressing this challenge. These models typically involve care managers, psychiatric consultation, and systematic follow-up protocols. Stepped-care models provide a structured way to manage depression by aligning the level of intervention with the severity of symptoms. Mild to moderate cases can be addressed initially by cardiologists or primary care clinicians using options such as antidepressants (notably selective serotonin reuptake inhibitors {SSRIs}), cognitive-behavioral therapy, or structured exercise programs. Patients with more complex needs, inadequate response, or severe depression can then be escalated to multidisciplinary collaborative care teams or specialized psychiatric services [26].

Implementation Barriers

Systematic evaluation of implementation barriers identifies multiple system-level challenges, including time constraints, inadequate workflow integration, and a lack of ownership of depression screening responsibilities. Healthcare providers report time constraints, failure to attend to all protocol steps, and lack of education and feedback as important implementation barriers. A critical system-level challenge involves mental health resource availability, with over 35% of the U.S. population residing in mental health professional shortage areas. Patients screening positive may face extended wait times or inability to access mental health services, undermining the utility of screening without adequate treatment infrastructure [27,28].

Integration challenges extend to electronic health record systems, referral processes, and communication between cardiology and mental health providers. Providers identify needs for standardized screening tools, clear clinical directions for different depressive symptom classifications, and formal protocols integrated into routine workflow. Provider-level barriers include insufficient knowledge to recognize depression and its cardiovascular connections, lack of time to conduct screening and initiate referrals, and competing clinical priorities. Symptoms of depression, including fatigue, weight loss, poor appetite, and sleep disturbance, overlap with cardiac disease manifestations, leading to attribution of depressive symptoms to physical illness [29].

Healthcare providers report feeling uncomfortable or not responsible for addressing patients’ mental health concerns. Historical separation of mental health from cardiology practice contributes to this discomfort. Additionally, providers express concerns about patient stigma associated with mental health diagnoses and treatment. Patient-level barriers encompass inadequate knowledge about depression and its treatment, mobility limitations affecting access to mental health services, and symptom denial or minimization. Qualitative research with cardiac patients reveals that timing of screening and referral represents a significant barrier, with patients reporting needs for longer-term follow-up beyond immediate post-event periods. Lack of information provision and understanding about mental health, especially following cardiac events, further impedes uptake of services. Stigma associated with mental health diagnosis and treatment remains a persistent barrier. Cultural factors, health literacy limitations, and previous negative experiences with mental health services contribute to low treatment acceptance rates. Insurance coverage limitations and out-of-pocket costs for mental health services present additional patient-level barriers, particularly in underserved populations [30,31].

Discussion

This narrative review synthesizes substantial evidence supporting the clinical importance and feasibility of depression screening in cardiology clinics. The high prevalence of depression in cardiac populations (15-30%), combined with its adverse impact on cardiovascular outcomes, quality of life, and healthcare utilization, provides a strong rationale for systematic screening. The availability of brief, validated screening instruments with acceptable psychometric properties (PHQ-2/PHQ-9) addresses concerns about time burden and diagnostic accuracy. However, the evidence also reveals significant implementation challenges that must be addressed for screening programs to achieve their potential benefits. The gap between screening recommendations in guidelines and actual implementation in practice, combined with inadequate follow-up of positive screens in many settings, demonstrates that successful programs require more than simply administering questionnaires. Evidence regarding the impact of systematic depression screening on patient outcomes in cardiac populations demonstrates mixed results. While screening consistently increases depression detection rates, the critical question involves whether increased detection translates into improved treatment engagement and clinical outcomes. Studies conducted in cardiac routine care demonstrate that patients with positive depression screens frequently do not receive adequate follow-up care, highlighting significant gaps in the detection-to-treatment pathway. This finding underscores that screening alone, without robust systems for subsequent treatment and follow-up, provides limited benefit. When patients are screened per protocol and positive cases identified, clinical response occurs in approximately 90% of cases, suggesting that the primary challenge lies in ensuring consistent screening rather than response to identified cases. This emphasizes the importance of implementation fidelity [32].

While additional data are needed regarding the effects of routine depression screening on cardiovascular outcomes, active engagement of cardiologists in screening may reduce stigma associated with depression and improve patient quality of life. Evidence from randomized controlled trials of antidepressant treatment for depressed cardiac patients shows no advantage regarding cardiovascular outcomes, though improvement in depressive symptoms, regardless of method, may lead to reduced subsequent cardiovascular events. This nuanced evidence base suggests that the primary benefit of screening lies in the identification and treatment of depression as a condition affecting quality of life and functional status, rather than direct cardiovascular risk reduction. However, indirect benefits through improved medication adherence, increased cardiac rehabilitation participation, and healthier lifestyle behaviors may contribute to better cardiovascular outcomes [33].

Recommendations for Practice

Based on the synthesized evidence, several recommendations emerge for cardiology practices seeking to implement or improve depression screening, as follows: adopt a systematic two-step screening protocol, such as implementing the PHQ-2/PHQ-9 two-step approach recommended by the American Heart Association and other cardiovascular organizations. This protocol balances efficiency with diagnostic accuracy while including assessment of suicidal ideation [24]. Integrate screening into routine workflow by utilizing electronic health record systems to prompt screening at appropriate intervals, automatically expand PHQ-2 to PHQ-9 when indicated, and trigger clinical decision support for positive screens, while designating clear roles and responsibilities for screening administration, interpretation, and follow-up [20]. Establish mental health partnerships by developing collaborative relationships with mental health providers through integrated care models, formal referral partnerships, or telemedicine arrangements, and ensure that screening is coupled with accessible pathways to evidence-based treatment [25]. Provide provider education by educating cardiology providers about depression-cardiovascular disease relationships, screening protocols, interpretation of results, and appropriate responses, including referral processes, while addressing concerns about the scope of practice and providing ongoing support and feedback [26]. Engage patients as partners by discussing the importance of depression screening prior to administration to improve acceptability, providing education about depression symptoms, the depression-heart disease connection, and available treatments, and addressing stigma through normalization and emphasis on depression as a medical condition affecting cardiac prognosis [31]. Monitor implementation fidelity by regularly assessing screening rates, positive screen identification, follow-up completion, and treatment initiation, and use quality metrics to identify gaps and guide continuous improvement efforts [33]. Address system-level barriers by allocating resources for screening programs, including staff time, electronic health record optimization, and mental health service access, and consider collaborative care or stepped-care models that integrate mental health professionals into cardiology practice [30].

Strengths of the review

The review systematically evaluates various depression screening instruments (PHQ-9, HADS, BDI) and analyzes their psychometric properties, feasibility, and applicability in cardiac populations, helping clinicians choose appropriate tools. It synthesizes findings from 66 studies to provide clear, evidence-based guidance on implementing depression screening protocols in cardiology clinics, including recommendations on timing, frequency, and follow-up procedures. Also, the review explores the complex interplay between depression and cardiovascular disease, acknowledging both the increased prevalence of depression in cardiac patients and its impact on cardiovascular outcomes and treatment adherence.

Limitations and future directions

Several limitations of the current evidence base warrant acknowledgment. First, the majority of studies focus on screening in English-speaking, high-income countries, limiting generalizability to diverse international contexts. Validation of screening instruments in various languages and cultural settings requires ongoing attention.

Second, long-term outcome studies examining the impact of systematic screening programs on cardiovascular events, mortality, and quality of life remain limited. While the rationale for screening is strong, based on depression prevalence and established depression-CVD relationships, definitive evidence that screening programs improve cardiovascular outcomes awaits further investigation.

Third, optimal screening intervals and populations require better definition. While guidelines recommend screening during hospitalization and at regular post-MI intervals, specific timing and duration of screening protocols need empirical validation. Questions about which cardiac populations benefit most from screening (e.g., post-MI, heart failure, stable coronary artery disease {CAD}) and whether universal screening or targeted approaches are most effective remain unanswered.

Future research should develop and evaluate innovative interventions addressing multilevel barriers while actively involving patients as partners in depression care. These interventions should be developed through theory-driven, transparent, multistage processes involving key stakeholders, including patients, nurses, and cardiologists, with rigorous methodological evaluation to determine whether early depression detection leads to health benefits.

## Conclusions

Depression represents a prevalent and clinically significant comorbidity in cardiovascular patients that warrants systematic screening in cardiology practice. Evidence supports the feasibility and clinical value of implementing two-step screening protocols using the PHQ-2 and PHQ-9 instruments. However, successful implementation requires comprehensive strategies addressing multilevel barriers at system, provider, and patient levels. Collaborative care models that integrate mental health professionals into cardiology settings represent promising approaches to closing gaps between depression detection and treatment. Cardiology practices should prioritize the implementation of evidence-based depression screening protocols as part of comprehensive, patient-centered cardiovascular care.
